# On the Molecular Basis of D-Bifunctional Protein Deficiency Type III

**DOI:** 10.1371/journal.pone.0053688

**Published:** 2013-01-07

**Authors:** Maija L. Mehtälä, Marc F. Lensink, Laura P. Pietikäinen, J. Kalervo Hiltunen, Tuomo Glumoff

**Affiliations:** 1 Department of Biochemistry and Biocenter Oulu, University of Oulu, Oulu, Finland; 2 Interdisciplinary Research Institute, CNRS, Theoretical and Computational Molecular Biology, Villeneuve d’Ascq, France; National Research Council of Italy, Italy

## Abstract

Molecular basis of D-bifunctional protein (D-BP) deficiency was studied with wild type and five disease-causing variants of 3R-hydroxyacyl-CoA dehydrogenase fragment of the human MFE-2 (multifunctional enzyme type 2) protein. Complementation analysis *in vivo* in yeast and *in vitro* enzyme kinetic and stability determinants as well as *in silico* stability and structural fluctuation calculations were correlated with clinical data of known patients. Despite variations not affecting the catalytic residues, enzyme kinetic performance (K_m_, V_max_ and k_cat_) of the recombinant protein variants were compromised to a varying extent and this can be judged as the direct molecular cause for D-BP deficiency. Protein stability plays an additional role in producing non-functionality of MFE-2 in case structural variations affect cofactor or substrate binding sites. Structure-function considerations of the variant proteins matched well with the available data of the patients.

## Introduction

Peroxisomal disorders either arise from defects in peroxisomal biogenesis or are due to non-functional key enzymes of peroxisomal metabolism. D-bifunctional protein (D-BP) deficiency belongs to the latter category. Typically, a point mutation or a deletion is found in the gene *HSD17B4* coding for D-bifunctional protein (also known as multifunctional enzyme type 2; MFE-2), an enzyme responsible for the second and the third reactions of the four-step fatty acid β-oxidation spiral in peroxisomes. MFE-2 is able to use very long straight-chain substrates, α-methyl-branched chain fatty acids and C27-bile acid intermediates [1], [2], which cannot be processed in mitochondria. Dysfunctional or residually active MFE-2 therefore leaves these types of lipids accumulating in cells. MFE-2 consists of two structurally distinct domains within a double-dimeric overall structure [3]: the 2E-enoyl-CoA hydratase 2 (“hydratase 2, H2”) and the 3R-hydroxyacyl-CoA dehydrogenase (“dehydrogenase, DH”) units. In the C-terminus of the human MFE-2, after the hydratase domain, there is a third domain consisting of an unspecific lipid-binding protein SCP-2L (sterol carrier protein type 2-like). This domain has no enzymatic activity and its precise function is unknown. All three functional domains of human MFE-2 can be studied as stand-alone proteins and their crystal structures are known [4]–[7].

D-BP deficiency results, via an unknown mechanism, in usually severe clinical abnormalities such as delayed psychomotor development, neonatal hypotonia and seizures, visual and hearing impairment, as well as craniofacial dysmorphic features [8]. Patients diagnosed with D-BP deficiency can be grouped into three groups: deficiency in both the hydratase and the dehydrogenase units (type I), the loss of activity of the hydratase unit of MFE-2 affecting the second reaction of β-oxidation (type II), or the loss of activity of the dehydrogenase unit of MFE-2 affecting the third reaction of β-oxidation (type III). The symptoms are the same regardless of the type of deficiency [9]. The clinical manifestations of D-BP deficient patients are similar to those of patients affected by a peroxisome biogenesis disorder collectively called the Zellweger spectrum disorders. Diagnosis of the deficiency is complemented by measurements of the levels of indicative fatty acids in plasma, fatty acids and enzyme activities in patient’s cells, usually skin fibroblasts, and mutation analysis. Analyses have revealed both missense and nonsense mutations with varying effects on the protein structure and residual activity of either or both enzymatic domains of MFE-2 [10].

We have previously studied a cohort of 110 D-BP deficiency patients with clinical and biochemical data available [9]. Several of these patients presented milder symptoms and extended life span. *In silico* protein structural studies indicated a correlation between the severity of the disease and the degree of disturbance to the protein structure. In this paper we report further structure-function studies with the aim to understand the molecular basis and the mechanisms leading to D-BP deficiency. Based on our previous studies all functional domains of human MFE-2 can be expressed and purified as stand-alone proteins that fold into their native conformations as fully active dimers [5]–[7]. When dimerization occurs the contacts in full-length MFE-2 are mainly between enzyme units of different monomers rather than within a monomer [7]. In this study we only focused on the variations located in the dehydrogenase domain to study the activity-stability relationship *in vitro* and *in silico*. Experimental structure, stability and activity data of the MFE-2 dehydrogenase domain is correlated with molecular dynamics (MD) simulation data of the protein showing the altered structural dynamics of the disease-causing variants with respect to the wild type enzyme.

## Materials and Methods

### Preparation of Protein Samples

The cDNA of the 3R-hydroxyacyl-CoA dehydrogenase (DH) fragment of HsMFE-2 [11] was amplified using PCR with a primer pair of 5′-tattatacatATGGGCTCACCGCTGAGGTTC-3′/5′-tattactcgagTGCTGTAGACGTTGCACGAC-3′ with lowercase sequences indicating mismatches to the HsDH sequence and NdeI and XhoI restriction enzyme recognition sites underlined. The codon usage of HsMFE-2 template was optimized for E. coli to improve the recombinant protein expression: altogether 15 codons (13 arginines and 2 isoleucines) were exchanged while keeping the native amino acid sequence intact (Table S1). The DH fragment was subsequently cloned into a pLWRP51-vector [12] by digesting the *Hs*DH insert and the pLWRP51 vector similarly with NdeI and XhoI restriction enzymes and then ligated by using a Fast-Link DNA Ligation Kit (Epicentre) resulting in pLWRP51::*HsDH* bacterial expression plasmid. The nucleotide sequence of the inserted DNA was checked for possible mutations.

The plasmid was used as a template in PCR for constructing all of the patient variants [9] with *HsDH*-spesific primers using QuickChange™ site-directed mutagenesis kit (Stratagene). Mutagenesis was carried out according to the instructions. The expression and purification of *Hs*DH and the patient variants were done as follows. The pLWRP51::*HsDH* plasmids were transformed into *E. coli* BL21 (DE3) pLysS competent cells (Novagen). Selection was done in LB-ampicillin-chloramphenicol plates. Protein expression was done in M9ZB liquid medium (1% casein hydrolysate (Sigma), 90 mM NaCl, 1 mM MgSO_4_, 0.4% dextrose, 20 mM NH_4_Cl, 20 mM KH_2_PO_4_, 20 mM Na_2_HPO_4_) supplemented with carbenicillin (to 50 µg/ml) and chloramphenicol (to 34 µg/ml). Freshly grown colonies were picked from LB-amp-chloramphenicol plates and grown in a flask containing 1 liter of medium at +37°C until an OD_600_ of 0.8 was reached. The temperature was decreased to +18°C and the expression of the recombinant protein was induced by the addition of IPTG to a final concentration of 0.6 mM. After 16 h of induction, the cells were collected by centrifugation (4000 g) at +4°C for 30 min, and washed with PBS buffer. The cell pellet was stored at –70 °C until used.

The bacterial cell pellet (∼5 g wet weight) was resuspended in 40 ml of Lysis buffer (50 mM NaPi, pH 8.0, 300 mM NaCl, 5% glycerol). The cell wall was broken with lysozyme (to 300 µg/ml) at RT for 20 min and by sonicating 6 × 20 s, with 20 s resting intervals, in plastic tubes cooled on ice using a Soniprep 150 Ultrasonic Disintegrator (Sanyo Gallenkamp PLC). The suspension was subsequently centrifuged (10,000 g) at +4°C for 45 min and filtered through glass wool. The supernatant was applied to a Ni–NTA Superflow -column (0.75 cm × 1.0 cm, Qiagen) equilibrated with the Lysis buffer. Unbound proteins were washed off the column with the same buffer and recombinant protein was eluted from the column by a 70 ml linear gradient of 0–250 mM imidazole (pH 8.0). The flow rate in every step was 1 ml/min. The fractions containing recombinant protein were pooled and concentrated by using an Amicon® Ultra Centrifugal Filter Devices (Millipore) with a cut off 5.0 kDa. The concentrated protein sample was applied in a 350 µl aliquot to a Superdex 200 10/300 GL size-exclusion column (GE Healthcare) equilibrated in PIPES/HCl buffer (20 mM piperazine-N,N′-bis(2-ethanesulfonic acid), pH 6.8, 150 mM NaCl, 1 mM EDTA, 5% glycerol, 1 mM NaN_3_) at 0.3 ml/min. Fractions (300 µl) containing pure wild type 3R-hydroxyacyl-CoA dehydrogenase recombinant protein or its clinically relevant variants were pooled and concentrated to 5–12 mg/ml.

### Enzyme Assays and in vivo Studies

The enzyme activities and kinetic constants were obtained by monitoring the oxidation of 3R-hydroxyacyl-CoA at 303 nm using a 2E-decenoyl-CoA substrate [13]. Concentration of the used substrate, synthesized as described by Qin *et al.* [14], was varied from 0.5 to 30 µM, measured by using Ellman’s test [15]. The reaction buffer included 50 mM Tris/HCl, pH 9.0, 50 mM KCl, 50 µg/ml BSA, 1 mM NAD^+^, 1 mM sodium pyruvate, 2.5 mM MgCl_2_, and 22 units lactate dehydrogenase (from rabbit muscle, Sigma) in a reaction volume of 0.5 ml. A surplus amount of recombinant pure *Hs*H2 protein [6] was used to convert the 2E-decenoyl-CoA into the substrate for *Hs*DH. Kinetic constants K_m_, V_max_ and k_cat_ were calculated by using the GraFit 5 program (Erithacus Software).

The yeast plasmids, pYE352::*HsMFE-2*, pYE352::*ScMFE-2* and pYE352::*CTA1*, used for *in vivo* experiments were constructed before [13], [16]. Plasmid pYE352::*HsMFE-2* was used as the template for patient variants *Hs*MFE-2(T15A), *Hs*MFE-2(N158D), *Hs*MFE-2(E232K), *Hs*MFE-2(R248C) and *Hs*MFE-2(W249G), using *HsDH*-spesific primers and QuickChange™ site-directed mutagenesis kit (Stratagene). All eight plasmids were transformed into *Saccharomyces cerevisiae* deletion strain BY4741 *Δfox2* (YKR009c, Euroscarf, accession code Y0508) (*Δfox2*) by the lithium acetate method [17] and selected on SD-Uracil (0.67% Yeast nitrogen base w/o amino acids, 0.2% Yeast synthetic drop-out media supplement w/o uracil, 2.0% dextrose, 2.0% agar) plates [16].

The yeast complementation studies were done by spotting the fresh o/n culture dilution series of ^1)^ *Δfox2*+ pYE352::*HsMFE-2*, ^2)^ *Δfox2*+ pYE352::*ScMFE-2*, ^3)^ *Δfox2* strain, ^4)^ *Δfox2*+ pYE352::*CTA1*, ^5)^ *Δfox2*+ pYE352::*HsMFE-2*(T15A), ^6)^ *Δfox2*+ pYE352::*HsMFE-2*(N158D), ^7)^ *Δfox2*+ pYE352::*HsMFE-2*(E232K), ^8)^ *Δfox2*+ pYE352::*HsMFE-2*(R248C), ^9)^ *Δfox2*+ pYE352::*HsMFE-2*(W249G) on thin oleic acid plates (0.67% yeast nitrogen base w/o amino acids, 0.1% Yeast extract, 0.5% KPi, pH 6.0, 0.5% Tween 80, 0.14% oleic acid, and 2.0% agar) [18]. For spotting assays the cells were washed with sterile water, the OD600s of washed cell cultures were normalized to 1.0, and the dilution series 10^−1^ to 10^−2^ were prepared in sterile water. The plates were incubated at 30°C for one week and an additional two days at 4°C for a better visualization of the clearing zones made by the cells able to utilize the oleic acid as the sole carbon source for growth. *ΔFox2* and *Δfox2*+ pYE352::*CTA1* were used as negative controls, *Δfox2*+ pYE352::*HsMFE-2* and *Δfox2*+ pYE352::*ScMFE-2* as positive controls.

### Circular Dichroism (CD) Spectroscopy

Far UV- circular dichroism spectra were recorded by a JASCO J-715 CD-spectropolarimeter (Jasco Corp. Tokyo, Japan) with the sample concentrations 0.050 mg/ml in 20 mM potassium phosphate, pH 7.0. All scans were collected at 25°C as an average of eight scans, using a cell with a path length of 0.1 cm and instrument settings: response 0.5 s, sensitivity 100 mdeg, and scan speed 20 nm/min. Thermal denaturation curves were generated by monitoring the protein solution at a fixed wavelength 222 nm over a temperature gradient of 25–90°C with the instrument settings: temperature increase 30°C/h, response 16 s, and sensitivity 100 mdeg.

### Molecular Dynamics Simulations

Wild type and variant structures were all prepared from Protein Data Bank [19] entry 1ZBQ [7]. We are assuming that variant structures do not differ too much from wild-type, which is a reasonable assumption for most point variations, corroborated by our experiments for at least 5 disease-causing variants. Since we consider it of prime importance to combine our simulation work with the available experimental data, we have chosen to omit those variants for which no such activity or stability data could be collected, moreover so since we have no evidence as to their stability and structural fold. The remaining 5 variants (plus the wild type structure) were prepared and investigated as follows. Missing side chain atoms were obtained from the other monomer if available, or else by copying the side chain conformation of a similar residue where the resulting conformation shows no overlap with neighboring residues. Residues were mutated by shaving off the side chain till the last common atom, and then renamed and rebuilt using the same procedure. All structures were placed in a truncated dodecahedron box and solvated with between 15760 and 15783 SPC [20] water molecules. All simulation systems were made electrostatically neutral by replacing the appropriate number of random water molecules with Cl^–^ -counter ions [21]. The Gromacs [22] suite of programs (version 3.3.1) was used, with the Gromos96 43a2 parameter set [23]. One thousand steps linear descent energy minimization was performed, applying harmonic position restraints (1000 kJ/mol/nm^2^) on the protein backbone atoms, followed by 100 ps position-restraint Molecular Dynamics (MD) simulation. A time step of 4 fs was employed, removing center-of-mass motion every time and updating the neighbor list every 5 steps. The systems were coupled to a temperature bath at 310 K using Berendsen temperature coupling, with a coupling constant of 0.1 ps [24]. Protein and solvent (water plus counter ions) were coupled separately. Pressure was maintained at 1 bar using isotropic pressure coupling with a coupling constant of 1 ps. Van der Waals interactions were cut off at a distance of 1.0 nm, electrostatic interactions calculated with the particle mesh Ewald method [25], using fourth-order splining and a grid spacing of 0.12 nm. Equations of motion for the water molecules were solved analytically with the SETTLE algorithm [26]. All bonds were constrained using the LinCS algorithm [27] and rotational motion involving CH_3_ groups was slowed down using virtual sites [28]. Production runs consisted of 50 ns simulation using the same settings, but without position restraining of the protein atoms. Our equilibration procedure included the first 20 ns of the production runs, when it was found that all simulations had reached equilibrium in terms of root mean square deviation and secondary structure evolution. Data collection and analysis is over the last 30 ns of every simulation, offering ample length to study the type of interactions and motions we were interested in. An exhaustive investigation of hydrogen bonding was performed, analyzing for every frame of the simulation trajectory the shortest distance between possible donor and acceptor atoms.

### Other Analyses

The concentrations of the protein samples were determined spectrophotometrically, measuring the absorbance at 280 nm and dividing it by absorbance coefficient [29]. Purities of the samples were analyzed by 12% SDS-polyacrylamide gels [30] and for the native *Hs*DH protein, using the static light scattering (SLS) as described previously [3].

## Results and Discussion

Solving crystal structures of protein variants including complexes with substrates, inhibitors or other ligands is the preferred method to study the molecular basis of enzyme function/dysfunction. Unfortunately, variant proteins are often difficult to express, purify and crystallize, due to the structural perturbation caused by the variation. Unlike many engineered proteins, amino acid substitutions as caused by mutations in patients frequently have an adverse effect on the activity or stability of the enzyme - otherwise they would not be revealed via a disease. Certain variant proteins do retain their structural integrity and activity, however, to the extent that they lend themselves for experimental molecular studies. This was also seen in the present study.

### Expression and Purification

**Figure 1:**
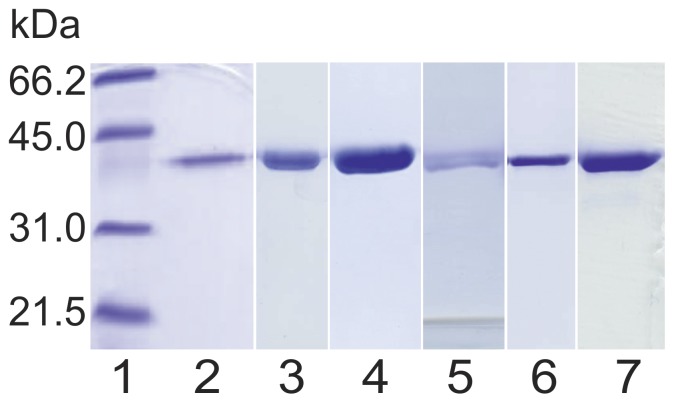
Coomassie-stained SDS-PAGE gel of purified *Hs*DH recombinant protein and its five clinically interesting patient variants under reducing conditions. The first line represents Low molecular weight protein standard (Bio-Rad) and lines 2–7 protein samples purified by Ni-NTA and Superdex 200 gel filtration columns as follows: 2) wild type *Hs*DH, 3) T15A variant, 4) N158D variant, 5) E232K variant, 6) R248C variant and 7) W249G variant. The molecular masses of all the recombinant protein monomers (∼36 kDa) correspond to the monomer mass (36.14 kDa) estimated from the aa sequence of the wild type *Hs*DH [29].

Ten clinically interesting and previously published [10] human 3R-hydroxyacyl-CoA dehydrogenase domain variants found by genetic analysis were chosen for the study. All variant proteins could be expressed and detected in *E. coli*. However, five of them could not be studied with respect to their enzymatic properties: *Hs*DH(A34V) turned out insoluble, two variants precipitated during the purification protocols (*Hs*DH(F237S) and *Hs*DH(A241T)), while two rapidly degraded after purification (*Hs*DH(R104M) and *Hs*DH(S153L)). Finally, the native *Hs*DH and the variants *Hs*DH(T15A), *Hs*DH(N158D), *Hs*DH(E232K), *Hs*DH(R248C) and *Hs*DH(W249G) were overexpressed in a pLWRP51 expression vector in *E. coli* BL21(DE3)pLysS cells. All recombinant proteins were purified chromatographically on a Ni-NTA column followed by a size-exclusion (Superdex 200) column. Five grams (wet weight) of *E. coli* cells provided 5 to 10 mg batches of pure recombinant proteins. The molecular masses of the denatured proteins, determined using SDS-PAGE analysis, were approximately 36 kDa (Figure 1), which agrees with the monomer 36.14 kDa mass estimated from the amino acid sequence of the native dehydrogenase (ExPASy Bioinformatics Resource Portal). All purified proteins eluted from the size-exclusion chromatography with the same retention time which points to a similar oligomeric status. The SLS results show that the recombinant proteins are dimers (size 76 kDa), which is consistent with the data that full-length MFE-2 as well as its stand-alone enzyme domains are known to be biologically active proteins only as dimers [5].

### Secondary Structures and Stabilities

The far-UV region (195–250 nm) CD spectra for native *Hs*DH and the five patient variants were virtually identical (data not shown), indicating a minor effect of the variations on the secondary structures and correct folding of the proteins. However, the spectrum of *Hs*DH(E232K) ran slightly more on negative values in wavelengths 205–222 nm. Secondary structure evolution in the MD simulations shows only a slight increase in the total number of secondary structure elements for variants R248C and W249G, and a change of balance between α-helices and turns for all variants (data not shown). Our results, both experimental and simulation, show only slight variations in secondary structure between the variant proteins, yet two of them suffer in stability.

Two of the variants, T15A and E232K, exhibited a lowered melting temperature (*T*_m_) of ca. 9°C (Table 1). A lowered *T*_m_ *in vitro*, although above the physiological temperature, is commonly used to indicate diminished stability of point variants also *in vivo*, and this has actually been shown for p53 [31]. E232K protein is a very active enzyme, while the T15A is clearly defective in terms of cofactor binding. In the former case the somewhat diminished activity is a sole consequence of diminished stability, while in the latter case the variation has hit a site crucial for both activity and stability. Thus, a lowered *T*_m_ alone is not a good indication of the disease-causing mechanism at the molecular level.

**Table 1:** Features of native recombinant *Hs*DH protein and its five clinically interesting patient mutation variants.

Protein	V_max_(µmol·min^−1^·mg^−1^)	K_m_(µM)	K_cat_(s^−1^)	K_cat_/K_m_	Tm(°C)	Structural contacts	Patients
NATIVE	4.44±0.21	0.54±0.11	1330	2460	58		
T15A	n.d.	n.d.	n.d.	n.d.	49	T15 is located in the NAD^+^ binding site. Hydrogen bondbetween side chain of T15 and main chain of N99 isbroken. This might disturb the structural feature, which iscommon to the SDR family members [32] and thecofactor binding groove could be loosened.	Three homozygote T15A patients having typical symptoms have been described, but the characteristic peroxisomal metabolites were normal or slightly increased/increased. One patient died in the age of 10 years and 9 months, others were still living at the ages of 8 and 5 [10], [34].
N158D	0.16±0.01	8.63±1.14	48.6	5.63	59	N158 is conserved residue (in mammalian and yeast MFE-2s) and locates in the substrate binding groove. According to docking experiments [35] it might be essential for the substratebinding via probably having a hydrogen bond with the substrate.This contact is most probably lost in the variant protein.	One heterozygote patient with the most common D-BP deficiency variation (G16S) has been mentioned [10].
E232K	2.59±0.21	0.38±0.11	777	2050	50	E232 forms a hydrogen bond with W273 from the othermonomer and takes part in the dimerization.Dimerization of the nucleotide binding domainand the extra C-terminal domains could be affected,leading to a more unstable structure.	One homozygote patient, whose age or peroxisomal metabolite levels are not known.
R248C	1.10±0.02	0.60±0.07	331	555	56	R248 locates in the area important for the substrate binding [33].	Three homozygote and one heterozygote (with W249G) patients have been mentioned previously [10]. One patient was still alive at the age of 5 and having normal levels of biochemical metabolites with the exception of elevated C26∶0 levels in plasma. The ratio of C26/C22 fatty acids was near normal.
W249G	0.019±0.001	2.68±0.47	5.7	2.1	58	W249 locates in the area important for the substratebinding [33]. W249s from both of the monomers pointinward in the crossing area of extra C-terminal domains andthey interact via strong π bond. Variation breaks astrong contact between two monomers and might disturbthe area important for the substrate binding.	One heterozygote (with R248C) patient has been mentioned previously [10]. Neither the age nor peroxisomal metabolite levels are known.

The activities of human dehydrogenase and its variants were measured by detecting spectrophotometrically the formation of magnesium complex of 3R-hydroxydecanoyl-CoA at 303 nm from the 2E-decenoyl-CoA substrate in concentration range from 0.5 to 30 µM. Kinetic parameters are calculated by using GraFit 5.0 program (Erithacus Software). Main features characterizing the observed structural changes in the variant proteins and the patients are also given (and discussed in more details in the text).

n.d. = not determined (recording reliable and comparable data would have necessitated using a large surplus of NAD^+^).

### Enzyme Activities

The kinetic parameters (Table 1) of the native and disease-causing variant *Hs*DH proteins were measured spectrophotometrically toward oxidation of 3R-hydroxydecanoyl-CoA, which becomes available for the dehydrogenase in the assay as a product of the hydratase 2 reaction. The results show differential residual activities for each of the proteins. For the T15A variant the location of the amino acid change in the binding site of NAD^+^ resulted in an inability to record reliable kinetic parameters by the method used. However, the *Hs*DH(T15A) variant undoubtedly had activity towards the 2E-decenoyl-CoA substrate used in the presence of a surplus of NAD^+^.

**Figure 2:**
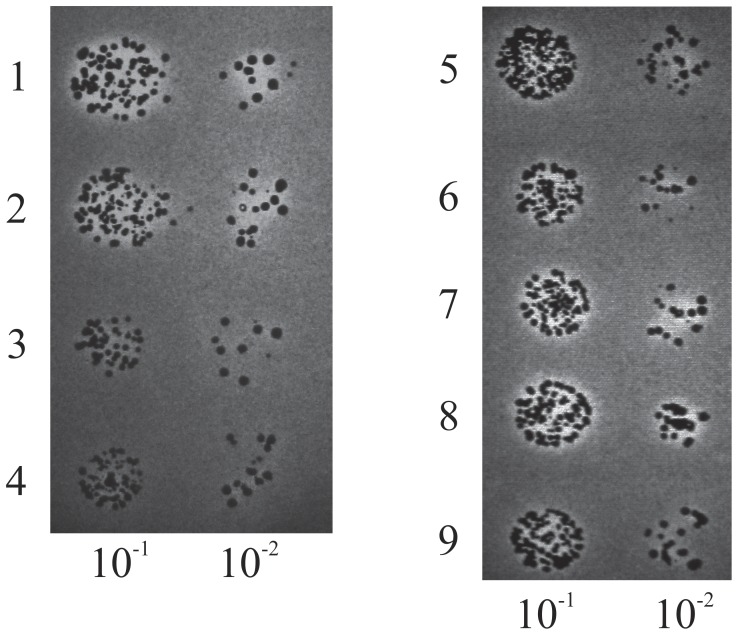
Growth of the BY4741 *Δfox2* cells on oleic acid transformed with pYE352::*ScMFE-2,* pYE352::*CTA1*, pYE352::*HsMFE-2*, and its five clinically interesting patient variants. The dilution series were done on YPD (0.2% glucose)- oleic acid (0.125%) plates and the BY4741 *Δfox2* cells were grown at +30°C for one week indicated by: (1) BY4741 *Δfox2*+ pYE352::*HsMFE-2*, (2) BY4741 *Δfox2*+ pYE352::*ScMFE-2*, (3) BY4741 *Δfox2* strain, (4) BY4741 *Δfox2*+ pYE352::*CTA1*, (5) BY4741 *Δfox2*+ pYE352::*HsMFE-2*(T15A), (6) BY4741 *Δfox2*+ pYE352::*HsMFE-2*(N158D), (7) BY4741 *Δfox2*+ pYE352::*HsMFE-2*(E232K), (8) BY4741 *Δfox2*+ pYE352::*HsMFE-2*(R248C), (9) BY4741 *Δfox2*+ pYE352::*HsMFE-2*(W249G). When the yeast is able to utilize the oleic acid as a sole source of carbon, there will appear a clear zone around (samples 1, 2, 5, 6, 7, 8 & 9), but if the functional *fox2* gene is missing the environment remains opaque (samples 3 & 4).

In the oleic acid experiment, utilizing *S. cerevisiae* lacking endogenous MFE-2, the ability of the full-length *Hs*MFE-2 variants for complementation was studied (Figure 2). In all cases the variant proteins rescued the ability of the yeast to utilize oleic acid as the sole carbon source, thus indicating *in vivo* activity of MFE-2 for all the studied variant proteins.

### Molecular Dynamics Simulations

It is not straightforward to analyze the influence of a point variation that removes a native hydrogen bond. If the donor or the acceptor atom is not present in the variant, the hydrogen bonding will occur to the nearest available protein atom or to a water molecule. Depending on the structural environment nearby or distant bonds may be affected.

Local disturbances to the structure are evident. However, our analyses revealed that gross structural changes affecting long distances over the protein molecule due to point variations are unlikely. We have performed an exhaustive investigation of internal hydrogen bonds and identified the existence of altogether 18 critically situated hydrogen bonds, distributed between e.g. substrate binding sites, the area of the catalytic triad and the C-terminal subdomain (Table 2). Since no clear evidence of domain movements in the variants were detected we decided to use the program HingeProt [32] to detect possible hinges and especially changes in hinge sites in order to reveal variation-specific domain movements in the simulated structures. For this purpose we performed a cluster analysis on the individual trajectories [33] using default parameters. The middle structure of the most populated cluster was subsequently analyzed with HingeProt. This was done for all six simulations as for the crystal structure. The analysis detected only minor changes with a hinge at residues 248/249 for all structures including the dehydrogenase crystal structure and a second hinge centering at residues 148, 167/171 or 189/190, which are all spatially close together lining the same inside of the substrate binding cavity. These findings confirm our observation that the point variations induce only a local disturbance in the structure as opposed to a global or long-range effect. Thus, we asked the more appropriate question whether an amino acid replacement in one way or another hampers local structural fluctuations in the protein molecule, more specifically the necessary dynamic movements associated with ligand binding or release, or catalytic activity. We then correlate these findings with our experimental assays and pathological data.

**Table 2:** The hydrogen bonds analyzed in MD simulations.

Atoms	Native	T15A	N158D	E232K	R248C	W249G	Note
**In the same monomer**							
T15 N - V97 O	+	−	+	+	+	+	NAD^+^ binding site
T15 OG1 - N99 N	+	-	−	−	−	−	NAD^+^ binding site
T15 OG1 - A100 N	+	-	−	−	−	−	NAD^+^ binding site
L103 N - F45 O	+	−	−	−	+	-	Above the catalytic triad (figure 4 A)
H123 NE2 - I102 O	+	+	+	−	+	-	Above the catalytic triad
Q141 NE2 - I93 O	+	−	+	+	−	+	On the surface
G160 O - R106 N	+	−	−	−	−	−	In the loops above the cavity
K168 NZ - T149 O	−	−	−	−	+	−	K168 belongs to the catalytic triad
E232 O - N190 ND2	+	+	+	−	+	−	N190 locates in Rossmann-fold β-sheet
R251 N - S283 O	+	+	−	+	+	+	Area important for the substrate binding (figure 4 A)
R251 O - S283 N	+	+	−	+	+	+	Area important for the substrate binding (figure 4 A)
**Between two monomers**							
N158 O - S177 OG	−	−	−	−	−	+	On the top of the cavity
E181 OE - F108 N	+	−	−	−	−	+	Above the cavity
E181 OE - G160 N	+	−	−	−	−	−	Above the cavity (figure 4 A)
R251 NH - E238 OE	-	−	+	−	−	−	Area important for the substrate binding
L253 O - W243 NE1	−	−	+	−	−	−	Catalytic domain and C-terminal subdomain dimerization
E267 OE - R32 NH	+	−	+	−	+	−	Catalytic domain and C-terminal subdomain dimerization (figure 4 A)
V257 N - G242 O	+	+	+	+	+	+	Catalytic domain and C-terminal subdomain dimerization

In total 18 hydrogen bonds, which were found to either be stable in the native protein or to form when the variation was present, were analyzed. The symbol (+) indicates the stable existence of the hydrogen bond and the symbol (–) clear fluctuation.

### Patient Variations

**Figure 3:**
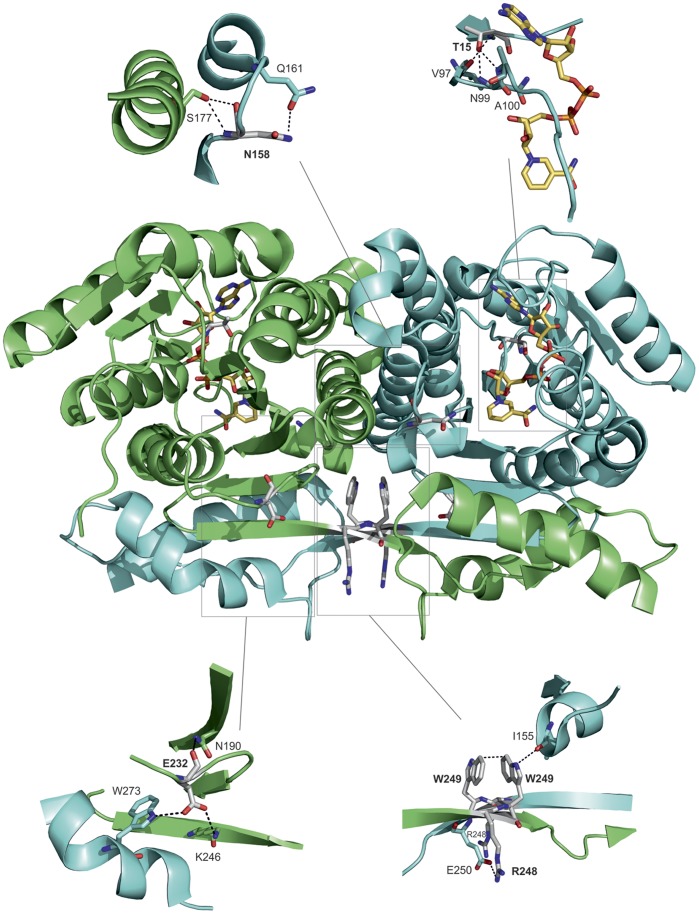
Five biologically interesting variations located in the dehydrogenase dimer of human MFE-2. The two dehydrogenase monomers in the middle of the figure are colored in green and blue, amino acids T15, N158, E232, R248 and W249 are shaded grey and shown in stick presentations, NAD^+^ are colored in yellow and shown also in stick presentation. The rectangles indicate parts of the structure that are represented in larger details in small figures. The figures were done using the program PyMol (Schrödinger) and human 3R-hydroxyacyl-CoA dehydrogenase structure (PDB ID 1ZBQ; [7]).

**Figure 4:**
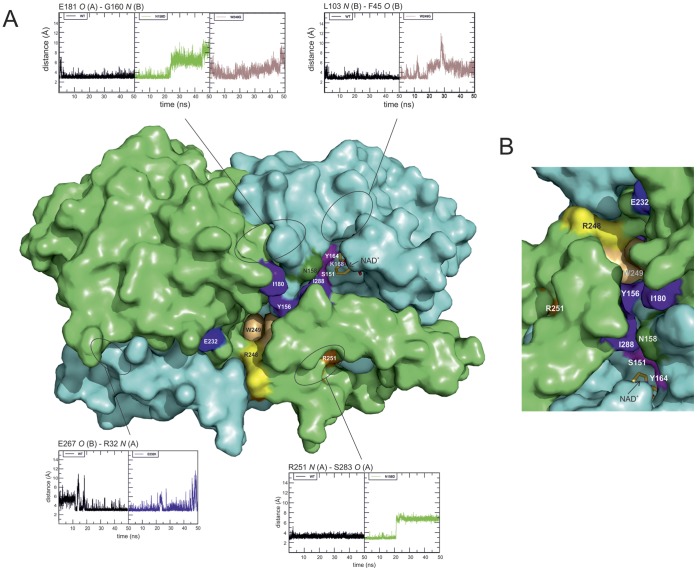
Hydrogen bond distance evolutions correlated with the variations. The two dehydrogenase monomers are shown as surface presentation; monomer A is colored in green and monomer B in blue. For both monomers the larger (upper) part in panel A is the Rossmann fold core domain and the smaller (lower) part is the C-terminal subdomain. Variation sites are color coded with the fluctuation analysis as follows: N158D (green), E232K (violet), R248C (yellow) and W249G (light brown). Residues found essential for discussion on the D-BP deficiency mechanism are colored as follows: amino acids belonging to catalytic triad (S151, T164 and K168) in magenta, amino acids locating at the tip of the cavity leading to the active site (I180, Y156 [36] and I288) in purple blue and amino acids important for the substrate binding (E250 and R251) in orange [36]. NAD^+^ is colored in yellow and presented in stick presentation. (A) The front side of both monomers. The groove for the substrate binding begins from the crossing area of C-terminal subdomains and continues to the cavity leading to the active site. Four different areas in which the variations cause structural fluctuation compared to native protein are marked with ellipses and linked to the fluctuation figures. In the fluctuation figures each rectangle shows the evolution of the distance (in nm; y-axis) of a specific hydrogen bond (as indicated) for 50 ns (x-axis). On each four fluctuation figures the leftmost rectangle (in black) is the native protein, while the other rectangles show the distance evolution of the indicated hydrogen bond in protein variants as follows: N158D (top left and bottom right in green), W249G (top left and right in light brown) and E232K (bottom left in violet). Representative surface-exposed hydrogen bonds were chosen for the figure; a complete list of hydrogen bonds, which were found fluctuating, is presented in table 2. (B) The substrate binding groove and cavity from above. The figures were done using the program PyMol (Schrödinger) and human 3R-hydroxyacyl-CoA dehydrogenase structure (PDB ID 1ZBQ).

Each amino acid residue change was introduced in the crystal structure coordinates of the native dehydrogenase (PDB code 1ZBQ; [7]) and the dynamics followed as described above. The rationale in our approach was to follow the existence and life-time of the hydrogen bonds present on or involving the residues affected by the variations. Hydrogen bonding distances (threshold 3.5 Å) and the distance fluctuations, identification of putative nearby replacing hydrogen bond partners, and similarity of the effects in both monomers of the dimeric protein were considered. This information, combined with experimental stability and activity of the native and variant proteins (Table 1), allowed us to make conclusions about the disease-causing mechanism of each variation. The structural details of variation sites are presented in Figure 3 and hydrogen bond distance evolutions as correlated with the variations are presented in Figure 4.

### T15A Variation

This residue change is located in the NAD^+^ binding site, where two β-strands critical for the binding environment are held together (Figure 3). It is known that a mutation causing the G16S substitution is the most common cause for type III D-BP deficiency [34] and it inactivates MFE-2 *in vivo* [14]. Hydrogen bonds connect the T15 side chain to the backbones of residues V97, N99 and A100. The bonds to N99 and A100 clearly break upon variation (distances increase from ca. 3Å to ca. 5Å). This could loosen the structure locally, which is supported by the increased temperature sensitivity (Table 1), and disturb the binding of NAD^+^, seen in the activity measurements as a need for a large surplus of NAD^+^ to achieve measurable data. In MD simulations the fluctuation extends also to the signature Ser-Tyr-Lys catalytic triad of the SDR superfamily [35], as well as to the cavity area (Figure 4) important for the substrate binding [36]. Most important seems to be, however, that the hydrogen bond not involving any side chain atoms (T15 *N* to V97 *O*) seems to be more stable. This is logical given the fact that the change of side chain does not directly affect this bond. It seems that this bond is enough to maintain the local environment sufficiently intact and the enzymatic activity is not completely lost: complementation assay in yeast indicates the ability of T15A-variated protein to utilize oleic acid (Figure 2) and activity towards 2E-decenoyl-CoA substrate was undoubtedly seen (although could not be reliably quantitated; Table 1).

Three patients carrying the T15A variation and showing the typical symptoms of peroxisomal disorders have been described previously [9], [37]. They all had normal or, after passing of time, slightly elevated peroxisomal parameters in plasma. One patient had normal C26∶0 β-oxidation in fibroblasts at the age of five, but pristanic acid β-oxidation was deficient. A boy, who passed away at the age of 10 years and 9 months, had slightly elevated pristanic acid level in plasma. A girl patient showed the increase of pristanic acid in plasma first time at the age of three and increase of C26∶0 and C26∶0/C22∶0 ratio at the age of 3.5 years. She was still living at the age of five. These cases together with our results suggest that the T15A variation results in D-BP deficiency of the mild type.

### N158D Variation

Despite the crystal structure indicating a firm hydrogen bond between N158 *ND2* and Q161 *OE1* (Figure 3) the MD simulations show that this connection is unstable in the wild type protein. In the variant protein this bonding is not even possible due to the lost hydrogen donor upon side chain alteration. The distances of nearby candidate dimerization contacts from S177 *OG* to either N158 *N* and/or N158 *O* increased notably in the MD simulations in the N158D variation compared to native one. It therefore seems that dynamicity is an inherent feature of this site and the variation *per se* should not increase the instability of the protein, as also seen in Table 1. Thus the observed loss of enzyme activity must follow from disturbance related to substrate binding, since N158 is one of the residues lining the substrate cavity (Figure 4) and is within binding distance to the 3R-hydroxydecanoyl substrate in the available docking structure [38]. This is consistent with the observation that the residue 158 is located in a loop lining the substrate cavity and its variation should have little effect on overall protein structure. Indeed, the *K*_m_ for this substrate is considerably affected (Table 1). However, yeast complementation experiment indicates at least some residual activity towards LCFAs (Figure 2). Altogether, in MD simulations this variation seems to cause fluctuations in the Ser-Tyr-Lys catalytic triad area [39], in the area of substrate cavity as well as in the crossing area of the two C-terminal subdomains (Figure 4) (see also section *R248C and W249G variations*). These two latter areas have been shown to be essential for substrate binding also by Kashiwayama *et al*. [36] with N158D variation causing decreased capability to bind VLCFA substrates. Also the NAD^+^ binding site seems to have increased fluctuation. Structurally N158 is located centrally to all these essential areas for substrate binding and activity of the protein (Figure 4) and therefore we can conclude that the correct placing of the substrate is compromised. The low measured dehydrogenase activity matches with the early decease age of a known patient (Dr. Sacha Ferdinandusse, personal communication).

One heterozygote N158D patient with the most common G16S variation has been reported previously [9]. The full-length protein and both dehydrogenase and hydratase 2 proteins were detected in immunoblots, but there is no information on biochemical metabolites. The patient passed away at the age of 14 months (Dr. Sacha Ferdinandusse, personal communication). The most common variation G16S is severe causing the decease before the age of two of the homozygote patients [9]. Our results indicate that N158D variation also causes the severe form of D-BP deficiency and probably results in impaired capability of utilizing LCFAs/VLCFAs.

### E232K Variation

E232 resides in a loop and its side chain takes part in a salt bridge to K246 within the monomer and relays a dimerization contact to W273 in the other monomer (Figure 3). These contacts are obviously a very labile part of the structure since the above bonds show large distance fluctuations in the simulations in the native enzyme as well as in all the studied variations. The backbone of E232 forms a hydrogen bond with N190 located in one of the Rossmann fold β-sheets [40] within the monomer. This bond seems to be stable in the native structure but disturbed by E232K variation (distance from 3 Å to 7 Å). However, 232 site is distant from the substrate binding site (Figure 4) and therefore an increased local dynamicity may not affect enzymatic properties as much as it may affect stability. The MD simulations show the E232K variation to cause more hydrogen bond fluctuations over the whole protein than the other studied variants: possible affected sites include areas essential for activity and dimerization. The effects do not seem to be so severe that the activity of the recombinant protein would be decreased dramatically and this variant probably is a decent enzyme with the efficiency (k_cat_/*K*_m_) towards MCFAs remaining at 83% of the native dehydrogenase (Table 1). In addition, the oleic acid experiment shows capability to utilize LCFAs (Figure 2). However, stability of this variant is reduced, both *in vitro*, as the recombinant protein is more prone to denaturation with ca. 9°C reduced thermal stability (Table 1) and *in vivo*, as the full-length, dehydrogense and hydratase 2 proteins are almost invisible in the immunoblots from patient fibroblasts (Dr. Sacha Ferdinandusse, personal communication). Unfortunately, no other data concerning this E232K homozygote patient is known, but it is clear that E232K variation causes D-BP deficiency by a mechanism related to instability of the protein.

### R248C and W249G Variations

These adjacent variations are convenient to discuss as a group. They are located at a characteristic crossing of β-strands (Figure 3) connecting the Rossmann fold core domain and the C-terminal subdomain of each dehydrogenase (or MFE-2) monomer, but at the same time provide a dimerization contact in the native enzyme [5]. We recall at this moment that the main hinge motion in the protein structure is inter-dimeric and centering around residues 248/249. There are no double variants known, however, so the structural, enzymatic and stability changes of both variations can conveniently be compared.

The R248 *NH* - E250 *OE* bond (Figure 3) appears to be unstable in the native enzyme, possibly due to its position at the outer rim of a potentially dynamic area. Other feasible bonds are not seen in the crystal structure. The R248C variation seems to cause fluctuation in the Ser-Tyr-Lys catalytic triad area, in the cavity area as well as in NAD^+^ binding area, but the increase in the interatomic distances is mostly small compared to other variations. It is therefore likely that R248 cannot be one of the most crucial dimerization contacts, and therefore variation in this site is not enough to totally abolish the enzyme activity (Table 1). This view is supported by the available data on a patient whose age was over five years [9].

W249, in turn, forms a π-stacking interaction with the same residue from the other monomer (Figure 3). It is conceivable that the W249–W249 stacking provides a more firm dimerization connection at this site than R248. Stacking interactions in proteins and protein-DNA complexes involving aromatic groups typically range between 20 and 45 kJ/mol [41], [42], while a single hydrogen bond between an amide and a carbonyl group remains at ca. 12.5 kJ/mol [43]. Losing the hydrogen bond at the 248 site perturbs the local structure less, since the site 249 stacking interaction remains, and therefore considerable enzyme activity and unaltered *K*_m_ (Table 1) are detected for R248C in comparison with W249G. On the contrary, if both tryptophans, which are directed towards the interior of the protein, are replaced by glycines, a large cohesive force is lost and a more probable outcome is a local opening of the dimeric structure and disturbance of the substrate binding, seen also as increased *K*_m_ and lost enzyme activity of W249G. It is therefore reasonable to conclude that W249 is at this site the dominant stability factor over R248.

In MD simulations W249G variation seems to cause fluctuation in the same areas as R248C: the cavity, the Ser-Tyr-Lys triad and the NAD^+^ binding area. Kashiwayama *et al.* [36] found that the area Trp249-Arg251 is essential for substrate binding and W249G variation leaves the capacity of the enzyme to bind substrates to a level of 30% of the native protein, a feature seen also in our results by increasing *K_m_*. In the yeast complementation experiment, both R248C and W249G enzymes could still utilize LCFAs (Figure 2).

Three R248C homozygote patients and one R248C-W249G heterozygote patient have been described previously [9]. The relatively long lifespan (>5 years) of one of the R248C patients, who was homozygote at the cDNA level but heterozygote at the genomic DNA level, indicates that R248C is a mild variation. On the contrary, the R248C-W249G heterozygote patient passed away in the age of 10 months. In the immunoblotting, the full-length MFE-2 and hydratase 2 protein levels of the heterozygote patient were decreased considerably and only a trace of dehydrogenase protein was seen (Dr. Sacha Ferdinandusse, personal communication). In light of these data it can be concluded that the virtually complete loss of enzyme activity due to the W249G variation possibly overrules the residual activity left by the R248C variation.

### Concluding Remarks

We have determined the enzyme kinetics and protein stability of the wild type and five disease-causing variations of the 3R-hydroxyacyl-CoA dehydrogenase fragment of human MFE-2 *in vitro*. To shed light on the molecular basis of D-BP deficiency we have used molecular dynamics simulation to produce ensembles of variant protein structures, offering a dynamic view of the wild type protein and available patient variants. This approach appears to be a better tool than static structural considerations in this case not even possible due to the lack of substrate-bound crystal structures of the disease-causing MFE-2 variants, to analyze consequences of specific lost chemical bonds in patient variants. A thorough analysis of hydrogen bonding involving variant residues and their surroundings was performed. The results correlated well with *in vitro* experimental data, with *in vivo* activity of the full-length MFE-2 as well as with clinical data of the patients.

Among the MFE-2 variants, amenable for overexpression and subsequent purification, V_max_ and the efficiency (k_cat_/*K*_m_) were significantly affected by variation an exception being E232K, however. Thus, the impaired enzyme kinetics can be concluded as a common denominator for the emergence of D-bifunctional protein deficiency in the set of variants studied. A complete loss of enzymatic activity probably leads to a severe form of the disease.

More interesting, however, is that the *in vitro* stability of the affected protein may not be a good indication of the severity of the disease. Reduced thermal stability of the T15A and E232K variations was not enough to abolish their activity, while the N158D and W249G variations result in inactive enzymes and severe form of the disease despite normal protein stability. It is noted that the affected stability and the opening of the structure (like e.g. domain movements or secondary structure fluctuations) are not necessarily connected. Important is whether this leads to impaired cofactor or substrate binding resulting in diminished catalytic efficiency, or whether the structural disturbance is distant from these sites with an outcome of minor dysfunction.

## Supporting Information

Table S1 **Changed rare amino acid codons in** ***HsMFE-2*** **cDNA.** In total 13 codons for arginine and 2 for isoleucine that are rare for *E. coli* were replaced with more common ones without changing the original amino acid sequence to increase the expression level of the recombinant *Hs*MFE-2 protein. The point mutations were done by using a QuikChangeTM site-directed mutagenesis kit (Stratagene).
(DOC)

## References

[1] Wanders RJ, Ferdinandusse S, Brites P, Kemp S (2010) Peroxisomes, lipid metabolism and lipotoxicity. Biochimica Et Biophysica Acta 1801: 272–280. 20064629 10.1016/j.bbalip.2010.01.001

[2] Russell DW (2009) Fifty years of advances in bile acid synthesis and metabolism. Journal of Lipid Research 50 Suppl: S120–5 10.1194/jlr.R800026-JLR200 PMC2674696 18815433

[3] Haataja TJ, Koski MK, Hiltunen JK, Glumoff T (2011) Peroxisomal multifunctional enzyme type 2 from the fruitfly: Dehydrogenase and hydratase act as separate entities, as revealed by structure and kinetics. The Biochemical Journal 435: 771–781. 21320074 10.1042/BJ20101661

[4] Haapalainen AM, van Aalten DM, Merilainen G, Jalonen JE, Pirila P, et al (2001) Crystal structure of the liganded SCP-2-like domain of human peroxisomal multifunctional enzyme type 2 at 1.75 Å resolution. Journal of Molecular Biology 313: 1127–1138. 11700068 10.1006/jmbi.2001.5084

[5] Haapalainen AM, Koski MK, Qin YM, Hiltunen JK, Glumoff T (2003) Binary structure of the two-domain (3R)-hydroxyacyl-CoA dehydrogenase from rat peroxisomal multifunctional enzyme type 2 at 2.38 Å resolution. Structure (London, England : 1993) 11: 87–97. 10.1016/s0969-2126(02)00931-0 12517343

[6] Koski KM, Haapalainen AM, Hiltunen JK, Glumoff T (2005) Crystal structure of 2-enoyl-CoA hydratase 2 from human peroxisomal multifunctional enzyme type 2. Journal of Molecular Biology 345: 1157–1169. 15644212 10.1016/j.jmb.2004.11.009

[7] Lukacik P, Shafqat N, Kavanagh K, Bray J, Von Delft F, et al. (2005) PDB ID: 1ZBQ. crystal structure of human 17-beta-hydroxysteroid dehydrogenase type 4 in complex with NAD.

[8] Wanders RJ, Barth PG, Heymans HSA (2001) Single peroxisomal enzyme deficiencies. In: Scriver CR, Beaudet AL, Sly WS, Valle D, editors. The molecular and metabolic bases of inherited disease. New York: McGraw-Hill. 3219–3256.

[9] Ferdinandusse S, Denis S, Mooyer PA, Dekker C, Duran M, et al (2006) Clinical and biochemical spectrum of D-bifunctional protein deficiency. Annals of Neurology 59: 92–104. 16278854 10.1002/ana.20702

[10] Ferdinandusse S, Ylianttila MS, Gloerich J, Koski MK, Oostheim W, et al (2006) Mutational spectrum of D-bifunctional protein deficiency and structure-based genotype-phenotype analysis. American Journal of Human Genetics 78: 112–124. 16385454 10.1086/498880 PMC1380208

[11] Qin YM, Haapalainen AM, Kilpelainen SH, Marttila MS, Koski MK, et al (2000) Human peroxisomal multifunctional enzyme type 2. site-directed mutagenesis studies show the importance of two protic residues for 2-enoyl-CoA hydratase 2 activity. The Journal of Biological Chemistry 275: 4965–4972. 10671535 10.1074/jbc.275.7.4965

[12] Alanen HI, Williamson RA, Howard MJ, Lappi AK, Jantti HP, et al (2003) Functional characterization of ERp18, a new endoplasmic reticulum-located thioredoxin superfamily member. The Journal of Biological Chemistry 278: 28912–28920. 12761212 10.1074/jbc.M304598200

[13] Hiltunen JK, Palosaari PM, Kunau WH (1989) Epimerization of 3-hydroxyacyl-CoA esters in rat liver. involvement of two 2-enoyl-CoA hydratases. The Journal of Biological Chemistry 264: 13536–13540. 2760034

[14] Qin YM, Marttila MS, Haapalainen AM, Siivari KM, Glumoff T, et al (1999) Yeast peroxisomal multifunctional enzyme: (3R)-hydroxyacyl-CoA dehydrogenase domains A and B are required for optimal growth on oleic acid. The Journal of Biological Chemistry 274: 28619–28625. 10497229 10.1074/jbc.274.40.28619

[15] Ellman GL (1959) Tissue sulfhydryl groups. Archives of Biochemistry and Biophysics 82: 70–77. 13650640 10.1016/0003-9861(59)90090-6

[16] Filppula SA, Sormunen RT, Hartig A, Kunau WH, Hiltunen JK (1995) Changing stereochemistry for a metabolic pathway *in vivo*. experiments with the peroxisomal beta-oxidation in yeast. The Journal of Biological Chemistry 270: 27453–27457. 7499202 10.1074/jbc.270.46.27453

[17] Gietz RD, Schiestl RH, Willems AR, Woods RA (1995) Studies on the transformation of intact yeast cells by the LiAc/SS-DNA/PEG procedure. Yeast 11: 355–360. 7785336 10.1002/yea.320110408

[18] Gurvitz A, Rottensteiner H, Kilpelainen SH, Hartig A, Hiltunen JK, et al (1997) The saccharomyces cerevisiae peroxisomal 2,4-dienoyl-CoA reductase is encoded by the oleate-inducible gene SPS19. The Journal of Biological Chemistry 272: 22140–22147. 9268358 10.1074/jbc.272.35.22140

[19] Berman HM, Westbrook J, Feng Z, Gilliland G, Bhat TN, et al (2000) The protein data bank. Nucleic Acids Research 28: 235–242. 10592235 10.1093/nar/28.1.235 PMC102472

[20] Berendsen HJ, Postma JPM, Van Gunsteren WF, Hermans J (1981) Interaction models for water in relation to protein hydration. Intermolecular Forces 11: 331–342.

[21] Chandrasekhar J, Spellmeyer DC, Jorgensen WL (1984) Energy component analysis for dilute aqueous solutions of Li^+^, Na^+^, F^-^, and Cl^-^ ions. Journal of the American Chemical Society 106: 903–910.

[22] Van Der Spoel D, Lindahl E, Hess B, Groenhof G, Mark AE, et al (2005) GROMACS: Fast, flexible, and free. Journal of Computational Chemistry 26: 1701–1718. 16211538 10.1002/jcc.20291

[23] Van Gunsteren WF, Billeter SR, Eising AA, Hünenberger PH, Krüger P, et al. (1996) Biomolecular simulation: The GROMOS96 manual and user guide. Zürich, Switzerland: Vdf. Hochschulverlag AG an der ETH Zürich.

[24] Berendsen HJ, Postma JPM, Van Gunsteren WF, DiNola A, Haak JR (1984) Molecular dynamics with coupling to an external bath. The Journal of Chemical Physics 81: 3684–3690.

[25] Essman U, Perera L, Berkowitz ML, Darden T, Lee H, et al (1995) A smooth particle mesh ewald method. The Journal of Chemical Physics 103: 8577–8592.

[26] Miyamoto S, Kollman PA (1992) SETTLE: An analytical version of the SHAKE and RATTLE algorithms for rigid water models. Journal of Computational Chemistry 13: 952–962.

[27] Hess B, Bekker H, Berendsen HJC, Fraaije JGEM (1997) LINCS: A linear constraint solver for molecular simulations. Journal of Computational Chemistry 18: 1463–1473.

[28] Feenstra KA, Hess B, Berendsen HJC (1999) Improving efficiency of large time-scale molecular dynamics simulations of hydrogen-rich systems. Journal of Computational Chemistry 20: 786–798. 10.1002/(SICI)1096-987X(199906)20:8<786::AID-JCC5>3.0.CO;2-B 35619462

[29] Gasteiger E, Gattiker A, Hoogland C, Ivanyi I, Appel RD, et al (2003) ExPASy: The proteomics server for in-depth protein knowledge and analysis. Nucleic Acids Research 31: 3784–3788. 12824418 10.1093/nar/gkg563 PMC168970

[30] Laemmli UK (1970) Cleavage of structural proteins during the assembly of the head of bacteriophage T4. Nature 227: 680–685. 5432063 10.1038/227680a0

[31] Mayer S, Rudiger S, Ang HC, Joerger AC, Fersht AR (2007) Correlation of levels of folded recombinant p53 in escherichia coli with thermodynamic stability in vitro. Journal of Molecular Biology 372: 268–276. 17631895 10.1016/j.jmb.2007.06.044

[32] Emekli U, Schneidman-Duhovny D, Wolfson HJ, Nussinov R, Haliloglu T (2008) HingeProt: Automated prediction of hinges in protein structures. Proteins 70: 1219–1227. 17847101 10.1002/prot.21613

[33] Daura X, Gademann K, Jaun B, Seebach D, van Gunsteren WF, et al (1999) Peptide folding: When simulation meets experiment. Angewandte Chemie International Edition 38: 236–240.

[34] van Grunsven EG, van Berkel E, Ijlst L, Vreken P, de Klerk JB, et al (1998) Peroxisomal D-hydroxyacyl-CoA dehydrogenase deficiency: Resolution of the enzyme defect and its molecular basis in bifunctional protein deficiency. Proceedings of the National Academy of Sciences of the United States of America 95: 2128–2133. 9482850 10.1073/pnas.95.5.2128 PMC19272

[35] Filling C, Berndt KD, Benach J, Knapp S, Prozorovski T, et al (2002) Critical residues for structure and catalysis in short-chain dehydrogenases/reductases. The Journal of Biological Chemistry 277: 25677–25684. 11976334 10.1074/jbc.M202160200

[36] Kashiwayama Y, Tomohiro T, Narita K, Suzumura M, Glumoff T, et al (2010) Identification of a substrate-binding site in a peroxisomal beta-oxidation enzyme by photoaffinity labeling with a novel palmitoyl derivative. The Journal of Biological Chemistry 285: 26315–26325. 20566640 10.1074/jbc.M110.104547 PMC2924054

[37] Gronborg S, Kratzner R, Spiegler J, Ferdinandusse S, Wanders RJ, et al (2010) Typical cMRI pattern as diagnostic clue for D-bifunctional protein deficiency without apparent biochemical abnormalities in plasma. American Journal of Medical Genetics.Part A 152A: 2845–2849. 20949532 10.1002/ajmg.a.33677

[38] Ylianttila MS, Pursiainen NV, Haapalainen AM, Juffer AH, Poirier Y, et al (2006) Crystal structure of yeast peroxisomal multifunctional enzyme: Structural basis for substrate specificity of (3R)-hydroxyacyl-CoA dehydrogenase units. Journal of Molecular Biology 358: 1286–1295. 16574148 10.1016/j.jmb.2006.03.001

[39] Jornvall H, Persson B, Krook M, Atrian S, Gonzalez-Duarte R, et al (1995) Short-chain dehydrogenases/reductases (SDR). Biochemistry 34: 6003–6013. 7742302 10.1021/bi00018a001

[40] Rao ST, Rossmann MG (1973) Comparison of super-secondary structures in proteins. Journal of Molecular Biology 76: 241–256. 4737475 10.1016/0022-2836(73)90388-4

[41] Churchill CD, Wetmore SD (2009) Noncovalent interactions involving histidine: The effect of charge on pi-pi stacking and T-shaped interactions with the DNA nucleobases. The Journal of Physical Chemistry.B 113: 16046–16058. 19904910 10.1021/jp907887y

[42] Copeland KL, Anderson JA, Farley AR, Cox JR, Tschumper GS (2008) Probing phenylalanine/adenine pi-stacking interactions in protein complexes with explicitly correlated and CCSD(T) computations. The Journal of Physical Chemistry.B 112: 14291–14295. 18922031 10.1021/jp805528v

[43] Schulz GE, Schirmer RH (1979) Principles of protein structure. New York: Springer-Verlag.

