# Supplementary material for: On the Molecular Basis of D-Bifunctional Protein Deficiency Type III
Source: PLoS One. 2013 Jan 7;8(1):e53688. doi: 10.1371/journal.pone.0053688 (PMC3538638; doi:10.1371/journal.pone.0053688)
Supplement: Table S1 — Changed rare amino acid codons in HsMFE-2 cDNA. In total 13 codons for arginine and 2 for isoleucine that are rare for E. coli were replaced with more common ones without changing the original amino acid sequence to increase the expression level of the recombinant HsMFE-2 protein. The point mutations were done by using a QuikChangeTM site-directed mutagenesis kit (Stratagene). (DOC) [file pone.0053688.s001.doc]

##### TABLE S1. Changed rare amino acid codons in *HsMFE-2* cDNA. In total 13 codons for arginine and 2 for isoleucine that are rare for *E. coli* were replaced with more common ones without changing the original amino acid sequence to increase the expression level of the recombinant *Hs*MFE-2 protein. The point mutations were done by using a QuikChangeTM site-directed mutagenesis kit (Stratagene).

| Amino acid | Rare codon | Frequency: per thousand [1] | Changed to | Frequency: per thousand [1] |
| --- | --- | --- | --- | --- |
| Arginine 6 | AGG | 1.4 | CGT | 24.1 |
| 10 | CGG | 4.6 | CGT | 24.1 |
| 23 | CGA | 3.1 | CGT | 24.1 |
| 32 | AGA | 2.1 | CGT | 24.1 |
| 63 | AGA | 2.1 | CGC | 22.1 |
| 64 | AGG | 1.4 | CGC | 22.1 |
| 65 | AGA | 2.1 | CGT | 24.1 |
| 92 | AGA | 2.1 | CGC | 22.1 |
| 110 | AGG | 1.4 | CGC | 22.1 |
| 121 | AGA | 2.1 | CGT | 24.1 |
| 145 | AGG | 1.4 | CGC | 22.1 |
| 183 | AGG | 1.4 | CGC | 22.1 |
| 258 | AGA | 2.1 | CGC | 22.1 |
| Isoleucine 93 | ATA | 4.1 | ATC | 26.5 |
| 111 | ATA | 4.1 | ATC | 26.5 |
|  | | | | |

References

1. Wada K, Wada Y, Ishibashi F, Gojobori T, Ikemura T. (1992) Codon usage tabulated from the GenBank genetic sequence data. Nucleic Acids Research 20 Supplement: 2111-2118.
